# Enhanced seed defenses potentially relax selection by seed predators against serotiny in lodgepole pine

**DOI:** 10.1002/ece3.6339

**Published:** 2020-05-09

**Authors:** Anna L. Parker, Craig W. Benkman

**Affiliations:** ^1^ Department of Zoology and Physiology University of Wyoming Laramie Wyoming USA; ^2^ Department of Biology University of North Carolina Chapel Hill North Carolina USA

**Keywords:** phenotypic selection, *Pinus contorta latifolia*, polymorphism, Rocky Mountains, seed predation, *Tamiasciurus*

## Abstract

Serotiny, the retention of seeds in a canopy seed bank until high temperatures cause seeds to be released, is an important life history trait for many woody plants in fire‐prone habitats. Serotiny provides a competitive advantage after fire but increases vulnerability to predispersal seed predation, due to the seeds being retained in clusters in predictable locations for extended periods. This creates opposing selection pressures. Serotiny is favored in areas of high fire frequency, but is selected against by predispersal seed predators. However, predation also selects for cone traits associated with seed defense that could reduce predation on serotinous cones and thereby relax selection against serotiny. This helps explain the elevated defenses in highly serotinous species. However, whether such interactions drive variation in seed defenses within variably serotinous populations has been studied rarely. We investigated the effects of phenotypic selection exerted by red squirrel (*Tamiasciurus hudsonicus*) predation on Rocky Mountain lodgepole pine (*Pinus contorta latifolia*) seeds. Squirrels preferentially harvested cones with more and larger seeds, indicating a preference for a higher food reward. We found evidence for stronger selection on trees with serotinous cones, which presumably accounts for the elevated defenses of and lower predation on serotinous compared to non‐serotinous cones. Lower levels of predation on serotinous cones in turn lessen selection against serotiny by squirrels. This has important implications because the frequency of serotiny in lodgepole pine has profound consequences for post‐fire communities and ecosystems widespread in the Rocky Mountains.

## Graphical abstract

Seed predation by red squirrels selects against serotiny (retention of seeds in a canopy seed bank), and thus, high predation pressure leads to the evolution of lower frequencies of serotiny. Here, we provide evidence that selection by red squirrels favoring more enhanced seed defenses in serotinous cones counters selection against serotiny by reducing the level of seed predation. This is of special importance because the frequency of serotiny influences the density of seedlings after a fire with massive community and ecosystem consequences.

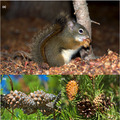

## 1 INTRODUCTION

Serotiny, the retention of seeds in a canopy seed bank, is an important life history trait of woody plants that are likely to experience a stand‐replacing fire (Lamont, Le Maitre, Cowling, & Enright, 1991). Accumulating and storing seeds in the canopy to be released after such a fire is highly advantageous, due to the abundance of resources and lack of competition (Causley, Fowler, Lamont, & He, 2016; Lamont et al., 1991). However, serotiny increases the opportunity for predispersal seed predators to harvest the seeds (Janzen, 1971; Lamont et al., 1991; Smith, 1970; Talluto & Benkman, 2014). This elevated risk of predispersal seed predation can counter and overwhelm selection from fire favoring serotiny (Talluto & Benkman, 2014; see also Enright, Marsula, Lamont, & Wissel, 1998). However, an elevated risk of predation should result in stronger selection for increased physical seed defenses and presumably explains why serotinous species allocate more resources to apparent seed defense (Smith, 1970; Janzen, 1971; Lamont et al., 1991; Groom & Lamont, 1997; Hulme & Benkman, 2002). This suggests that plants evolve seed defenses that counter selection by seed predators against serotiny. Whether the serotinous subpopulation within a variably serotinous population similarly experiences more intense selection for seed defenses and evolves elevated defenses relative to the non‐serotinous subpopulation, thereby relaxing selection by seed predators against serotiny, has not been addressed.

**FIGURE 1:**
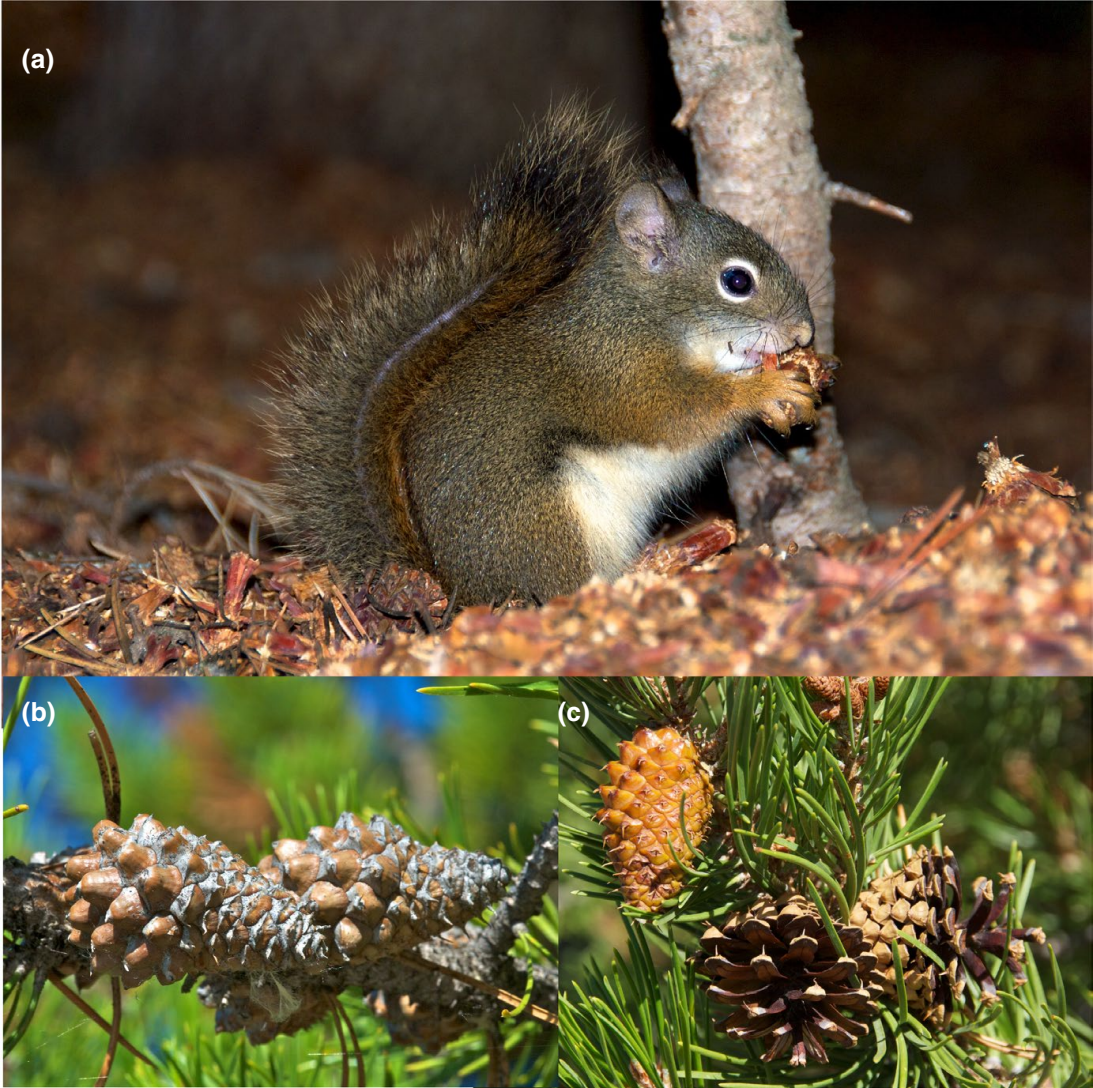
(a) A red squirrel (*Tamiasciurus hudsonicus*) eating a seed from a lodgepole pine (*Pinus contorta* ssp. *latifolia*) cone on top of a cone midden; only a few scales remain on the cone (on the distal end, directed downward). (b) Serotinous cones, which can remain closed for several decades unless removed by red squirrels or opened from heat of a fire. (c) Non‐serotinous cones open in early autumn several weeks after the seeds mature; leftmost cone opened within weeks after photograph was taken. From Benkman, Jech, & Talluto (2016) with permission. Photographs taken by C. Benkman

Here, we focus on Rocky Mountain lodgepole pine (*Pinus contorta latifolia*) and predispersal seed predation by red squirrels (*Tamiasciurus hudsonicus*). Young lodgepole pine initially produce almost exclusively non‐serotinous cones that open and release their seeds beginning in late summer, soon after maturation. After reaching about 30–50 years of age, some individuals shift to producing predominately serotinous cones (>90% of cones) that remain closed and hold seeds for years or decades, while others continue producing predominately non‐serotinous cones (Figure 1; Critchfield, 1980; Koch, 1996). Some older individuals produce intermediate numbers of the two cone types (10%–90% of either open or closed cones), but such trees are uncommon (Critchfield, 1980:8%–20% of individuals in 7 different lodgepole pine forests). At least some serotinous cones from some trees are susceptible to opening during unusually hot weather (Benkman, 2016; Critchfield, 1980). Such events will increase the occurrence of trees with mixed cone types, or with only open cones, and are likely to increase with climate change. However, the serotinous cones of most lodgepole pine are not as susceptible to cone opening under regularly experienced conditions as those of, for example, Aleppo pine (*P. halepensis*) (Nathan, Safriel, Meir, & Schiller, 1999), whose trees commonly have intermediate frequencies of closed cones (Martín‐Sanz et al., 2016).

Earlier work on lodgepole pine indicates that the frequency of serotiny, on both a local and regional scale, reflects a balance: Selection from increasing fire frequency favors higher frequencies of serotiny (Schoennagel, Turner, & Romme, 2003), whereas selection from increasing squirrel predation favors lower frequencies of serotiny (Benkman & Siepielski, 2004; Talluto & Benkman, 2013, 2014). At the highest squirrel densities, selection exerted by red squirrels against serotiny potentially overwhelms selection from high fire frequencies, resulting in nearly no serotiny (Talluto & Benkman, 2014). However, it is plausible that more intense selection exerted by seed predators on individuals producing serotinous cones leads to an increase in physical seed defenses that lessen the relative disadvantage experienced by individuals producing serotinous cones. The question, therefore, is whether evolution in response to selection by squirrels on seed defenses (besides non‐serotiny, which reduces predispersal predation through releasing seeds quickly after seeds mature) could act to disproportionately increase survival of serotinous cones, relaxing selection against serotiny. If this allows higher frequencies of serotiny than otherwise, then this is of special interest because pre‐fire frequency of serotiny has a tremendous impact on the density of lodgepole pine seedlings after a fire, and on many community and ecosystem attributes for decades afterward (Turner, Romme, & Tinker, 2003; Turner, Whitby, Tinker, & Romme, 2016).

Selection for greater resource allocation toward seed defense in serotinous cones is expected for at least two reasons related to the extended period available to harvest serotinous cones, as compared to the brief window of time that non‐serotinous cones are available (once cones begin opening, red squirrels cease harvesting them). First, the much greater opportunity to harvest serotinous cones can account for why red squirrels have been found to harvest a larger proportion of serotinous than non‐serotinous cones (Talluto & Benkman, 2014). This will lead to a greater opportunity for selection among individuals producing serotinous cones and result in more intense phenotypic selection for enhanced seed defenses, as long as the relationship between survival and cone traits is similar between individuals within each of the two subpopulations (Benkman, 2013; see also Queller, 2017: For a given covariance between relative fitness and phenotype, phenotypic selection is proportional to the reciprocal of mean [sub]population fitness). Second, red squirrels might be limited in their ability to sample non‐serotinous cones and to assess their relative value or profitability. If this leads to a less consistent relationship between survival and cone traits, then this will result in a further weakening of selection on the non‐serotinous subpopulation.

An association between serotiny and seed defenses assumes that a suite of cone traits can evolve independently between serotinous and non‐serotinous subpopulations. This is unlikely to be true, unless genes influencing serotiny and seed defenses are linked and inherited together (e.g., in linkage disequilibrium; Felsenstein, 1965). Such linkage could result from strong selection favoring an association. If the traits are not linked, independent assortment, random mating (lodgepole pine is wind pollinated), and recombination will prevent an association between serotiny and seed defense traits within a population of lodgepole pine. A genome‐wide association study of serotiny in lodgepole pine found 11 unlinked loci contributed to much of the variation in the occurrence of serotiny, with no genes of major effect (Parchman et al., 2012). Whether these loci influence seed defenses is unknown; cone traits are generally highly heritable in conifers (references in Benkman, Parchman, & Mezquida, 2010). Nevertheless, some evidence indicates that genes influencing serotiny and seed defenses are linked. In particular, Muir and Lotan (1985) found that serotinous lodgepole pine cones in western Montana had fewer full seeds (containing female gametophyte and embryo; hereafter kernel) and tended to have a lower ratio of seed mass to cone mass than those of non‐serotinous cones. These differences could reflect a stronger response to selection exerted by red squirrels on the serotinous subpopulation (Muir & Lotan, 1985), because red squirrels preferentially harvest lodgepole pine cones with more full seeds and higher ratios of seed mass to cone mass (Benkman, Holimon, & Smith 2001; Benkman, Parchman, Favis, & Siepielski, 2003; Elliott, 1974, 1988a; Smith, 1970).

Why red squirrels gain from a preference for lodgepole pine cones with both more and proportionately more seeds is related to the squirrel's cone caching behavior–they cache a single cone at a time, so the more seeds in a cone, the more energy cached per unit time–and to their foraging behavior (Elliott, 1988a; Smith, 1970). Red squirrels begin foraging by biting off the overlapping, hard, woody scales starting at the base of the cone (Figure 1). The scales in the lower half of a lodgepole pine cone lack full seeds, yet those scales need to be removed before squirrels can access seeds in the distal end. Because red squirrels bite through nearly all the scales and eat all the seeds, the number of seeds determines the reward and cone mass influences the cost of accessing seeds (Smith, 1970). Presumably because the ratio of seed mass to cone mass is strongly related to seed number (see Results), the time required to access an average lodgepole pine seed increases at an accelerating rate as the number of seeds per cone decreases (Elliott, 1988a). Thus, the ratio of seed mass to cone mass and seed number are inversely related to the extent seeds are defended (Benkman et al., 2001; 2003, 2010; Elliott, 1974, 1988a; Smith, 1970). Consistent with such an interpretation and with red squirrels having an evolutionary effect, both the ratio of seed mass to cone mass and the number of full seeds per cone average around 2.4 times greater in mountain ranges where lodgepole pine has evolved in the absence of red squirrels during the Holocene than in mountain ranges where red squirrels are common (Benkman et al., 2001).

The goals of our research can be divided into two parts. First, we quantified phenotypic selection exerted by red squirrels on serotinous and non‐serotinous cones. By recording predation on trees for which we quantified cone traits, we calculated the intensity of selection on various cone traits. Second, we determined whether traits differed between serotinous and non‐serotinous cones in a manner consistent with selection.

## 2 METHODS

### 2.1 Tree selection

In late May and early June 2017, we surveyed forested areas in Medicine Bow National Forest 1.6–3.4 km north of Foxpark, Wyoming (41.099°N, 106.141°W) to locate red squirrel territories. We searched for large squirrel middens–a central location within a territory where red squirrels store cones (Smith, 1968)–that were separated by >100 m from other large middens; this approximates an average squirrel territory size (0.56 ha, 130 km south southeast in the Front Range; Gurnell, 1984). We gathered data on 16 territories that showed recent squirrel activity (e.g., recently foraged‐on cones), had >20 trees whose canopies could be photographed and sampled for cones, and were farthest from human disturbance. We assume that the historic interval between fires in our study site is similar to that measured in lodgepole pine forests north and south of our study area (182 years [Kipfmueller & Baker, 2000]; 162–216 years [Sibold, Veblen, & González, 2006]). Based on both a mean interval between fires of 180–185 years and the simulation models for lodgepole pine in Talluto and Benkman (2014), the expected frequency of serotiny in our study area in the absence of red squirrels is ~0.45.

**FIGURE 2:**
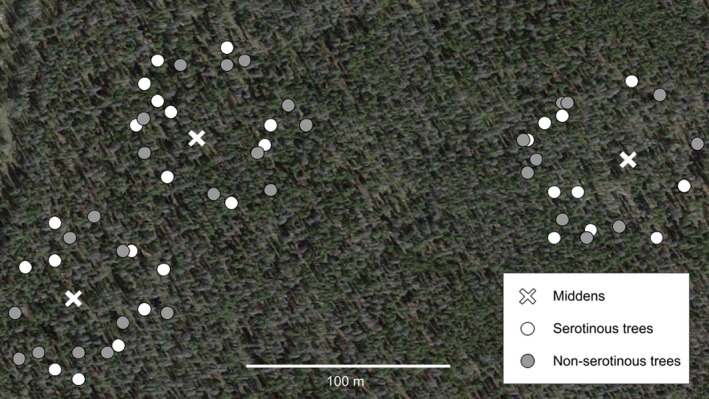
Aerial photograph showing locations of surveyed serotinous and non‐serotinous lodgepole pine in relation to the cone middens on three red squirrel territories

We haphazardly selected 10 trees with serotinous cones and 10 trees with non‐serotinous cones and a diameter‐at‐breast height (DBH) >10 cm within 15–40 m of each midden (Figure 2) for measuring cone survival and cone traits (mean ± SE distance from midden was 29.5 ± 0.8 m and 30.5 ± 0.9 m, respectively, for the two cone types; *t* test: *t* = 0.77, df = 125.8, *p* = .44). The mean DBH was 20.0 cm (*n* = 128 trees), indicating that most of the trees were >80 years old (Lotan & Critchfield, 1990). We chose this intermediate distance from the midden because closer to the midden all or nearly all the cones are harvested by squirrels, and farther away few cones are harvested (Talluto & Benkman, 2014). We chose trees if they had at least one clearly visible side for photographing cones and had only closed (serotinous) or open (non‐serotinous) older (>1 year old) cones. Approximately 20% of trees had both closed and open cones. These trees were excluded from analyses. This relatively high frequency of trees with a mix of closed and open cones is presumably related to our stringent criterion requiring all cones to be either closed or open (rather than >90%) for categorizing trees as serotinous and non‐serotinous, respectively (see Critchfield, 1980). We recorded distance from midden, as well as the GPS location of each tree, using a Garmin GPSMAP 64st. While searching for the above focal trees, we observed whether other mature trees were serotinous or not (35–40 trees per midden) to provide an approximate proportion of trees that were serotinous within 40 m of each midden.

### 2.2 Cone survival

Cone survival, defined as the proportion of cones remaining attached to a branch, was measured using photographs of the same branches over time (Talluto & Benkman, 2014). We photographed one side of each tree using a Nikon D200 camera and a Nikkor 80–400 mm telephoto lens during July 2017 to determine the number and location of developing cones. We hammered stakes into the ground at the location where photographs were taken, and returned to these locations to re‐photograph the trees. We attempted to re‐photograph the trees having non‐serotinous cones in October after all their cones would have opened, but we were only able to re‐photograph 12 of them due to adverse weather conditions. The remaining trees were re‐photographed in May 2018. We compiled the photographs using the program PanoramaStitcher (Version 1.10 (43); Boltnev & Kacher, 2018), thus creating one image for each tree. We counted only the developing cones (green, in contrast to the brown and gray of older cones) present in the July 2017 image. In the later images, the first‐year cones were counted as removed if their previous location was clearly visible and void of cones. Cones that could be clearly seen in both images were counted as surviving. If the location was obscured or out of focus in the 2018 image, the cone was treated as missing data and excluded from analysis. Because red squirrels are the only animals that remove Rocky Mountain lodgepole pine cones from the trees (Smith, 1970; Talluto & Benkman, 2014), and these cones otherwise remain attached to the branches “almost indefinitely” (Critchfield, 1957:57) with no apparent difference in cone retention times between open and closed cones (Muir & Lotan, 1985), these methods provide an estimate of cone survival with regard to squirrel predation. By limiting our analyses to first‐year cones, we underestimate the probability of predation a serotinous cone experiences because red squirrels can harvest such cones in subsequent years. However, this bias is likely small, because relatively few serotinous cones over a year old are harvested by red squirrels (Smith, 1970; Talluto & Benkman, 2014; but see Elliott, 1988b). We limited our analyses to the 129 trees (from the 312 trees photographed in July 2017) that had at least 3 developing cones whose locations were not obscured or out of focus in the second photograph taken in either October 2017 or May 2018.

### 2.3 Cone collection and measurement

We collected cones between 9 and 19 September 2017, because seeds and cones were mature, and non‐serotinous cones had not opened. We collected cones from each tree using a 20‐gauge shotgun to shoot off branches with cones. We then removed the cones from the branches, placed them in individually labeled plastic bags, and froze them. We shot cones from the side of the tree opposite where the photographs were taken for estimating cone survival, so that cone collection would not confound cone survival estimates. We attempted to collect three closed, mature cones from each tree, because the measurement of three cones generally provides an accurate estimate of the mean cone trait values for a tree (Garcia, Siepielski, & Benkman, 2009); within‐tree variation is usually much less than among‐tree variation for most cone traits (Garcia et al., 2009 and references therein). However, we were unable to collect three cones from 30 of the 129 trees (mean cone number per tree = 2.6; 1 cone from 16 trees; 2 cones from 14 trees; 3 cones from 99 trees); the smaller sample sizes for these 30 trees will act to increase the error in our estimation of their means. The number of cones measured did not differ between trees with serotinous and non‐serotinous cones (mean ± *SE*: 2.7 ± 0.08 and 2.6 ± 0.09, respectively; *t* test, *t* = 1.05, df = 127.0, *p* = .30).

We measured the length and width of each cone to the nearest 0.1 mm using digital calipers. Then, we removed moisture from the cones and opened them by placing them in a 60°C drying oven for 48–72 hr. All seeds were removed, and five full seeds were chosen, their seed coats removed, and the kernels weighed to the nearest 0.01 mg on a digital scale. We determined the number of full seeds by slicing through each remaining seed. Finally, the empty cone was weighed and the width of six scales, equidistant around the basal quarter of the cone, were measured. All analyses were conducted using mean tree values.

### 2.4 Data analysis

Selection gradients were estimated using multiple linear regression (Lande & Arnold, 1983). In addition to cone traits, we included diameter‐at‐breast height, distance from midden, proportion of trees having serotinous cones within 40 m of the midden, and Julian date of cone collection. Cone width to length ratio was used as a metric of cone shape. We standardized all trait values to a mean of 0 and a variance of 1 across all trees, and the response variable of survival was transformed to relative fitness by dividing by mean survival (Lande & Arnold, 1983). To avoid collinearity, we excluded variables with variance inflation factors > 2. Selection differentials were computed by conducting pairwise linear regressions between all standardized traits and relative fitness (Lande & Arnold, 1983) for trees with serotinous cones and for trees with non‐serotinous cones. We estimated the ratio of kernel mass to cone mass by multiplying the mean individual kernel mass by the number of full seeds divided by cone mass. We used a Kruskal–Wallis test to compare survival and *t* tests on ln‐transformed cone traits to quantify differences between trees with serotinous and non‐serotinous cones. All analyses were based on the 129 trees for which we had cone survival data. We conducted all analyses in JMP^®^ Pro 14.3.0.

## 3 RESULTS

**FIGURE 3:**
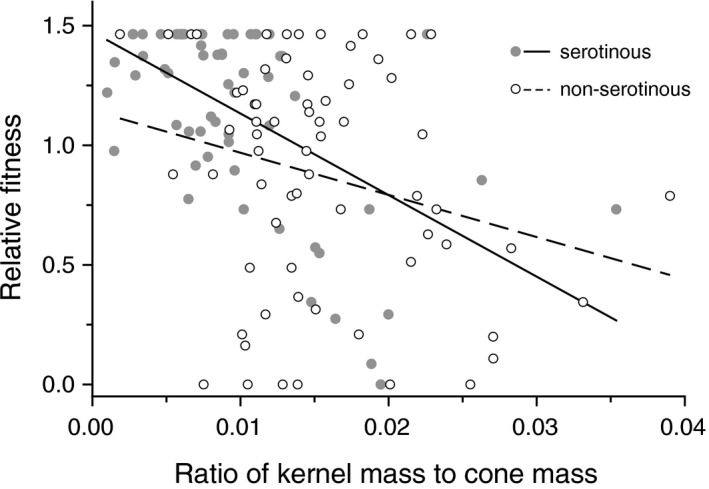
Relative fitness decreased more rapidly with increases in the ratio of kernel mass to cone mass in the serotinous than in the non‐serotinous subpopulation of lodgepole pine (see Table 2). The lines represent least squares linear regressions

Relative fitness decreased with increasing number of full seeds in the cone and tended to decrease with increasing size of kernels (Table 1), indicating that red squirrels preferentially harvested cones with more kernel mass (i.e., the “target” of selection). Such preferences resulted in negative selection (both direct and indirect selection) on the number of full seeds for trees of both cone types, and on kernel mass for trees with serotinous cones, but not for trees with non‐serotinous cones (Table 2). This suggests that selection by squirrels favors the evolution of smaller seeds in the subpopulation having serotinous cones but not in those having non‐serotinous cones. Selection was also detected on kernel to cone mass ratio in both cone types (Table 2; Figure 3). Because cone mass per se was not under selection (Tables 1 and 2), the selection on kernel to cone mass ratio was presumably the result of the strong correlation between the number of full seeds, which is a target of selection, and kernel to cone mass ratio (*r* = .80, *p* < .0001).

**TABLE 1:** Selection gradients for model that includes variables with variance inflation factors < 2

Trait	*β* ± *SE*	*p‐*value
Scale width	0.0253 ± 0.0407	.535
Cone width to length ratio	0.0086 ± 0.0376	.819
Kernel mass	−0.0777 ± 0.0394	.051
Number of seeds	−0.1835 ± 0.0407	<.0001
Cone mass	−0.0008 ± 0.0424	.984
Serotiny	−0.0458 ± 0.0401	.256
Tree diameter at breast height	0.0415 ± 0.0384	.282
Tree distance from the midden	−0.0085 ± 0.0375	.820
Proportion of trees with serotinous cones	0.0602 ± 0.0378	.114
Julian date of cone collection	−0.0388 ± 0.0377	.306

**TABLE 2:** Selection differentials (*β*′) for trees having either (A) serotinous cones or (B) non‐serotinous cones

Trait	*β*′ ± *SE*	*p‐*value
A *Serotinous cones*		
Scale width	−0.0090 ± 0.0544	.869
Cone length (mm)	−0.0701 ± 0.0504	.169
Cone width (mm)	−0.0249 ± 0.0491	.614
Cone width to length ratio	0.0420 ± 0.0460	.365
Number of full seeds	−0.1940 ± 0.0488	.0002
Individual seed kernel mass (mg)	−0.1319 ± 0.0435	.0036
Cone mass (g)	−0.0552 ± 0.0497	.271
Kernel mass to cone mass ratio	−0.2391 ± 0.0471	<.0001
B *Non‐serotinous cones*		
Scale width (mm)	−0.0359 ± 0.0551	.517
Cone length (mm)	−0.0455 ± 0.0576	.433
Cone width (mm)	−0.0376 ± 0.0596	.530
Cone width to length ratio	0.0116 ± 0.0639	.856
Number of full seeds	−0.1780 ± 0.0595	.0039
Individual seed kernel mass (mg)	−0.0079 ± 0.0634	.901
Cone mass (g)	0.0194 ± 0.0590	.743
Kernel mass to cone mass ratio	−0.1236 ± 0.0606	.0455

Serotinous cones had fewer seeds and a lower ratio of kernel to cone mass than non‐serotinous cones (Table 3). No other cone traits differed between serotinous and non‐serotinous cones (Table 3). The smaller kernel to cone mass ratio of serotinous than non‐serotinous cones is consistent with trees that have serotinous cones experiencing stronger negative selection on this trait (Figure 3; Table 2: the standard errors of the selection differentials did not overlap). Serotinous cones also tended to experience stronger negative selection on seed number than did non‐serotinous cones (Table 2), but it is unclear that such a trend alone is sufficient to account for the large difference in seed number between the two cone types (Table 3). In contrast, a greater difference in kernel mass than that found between the cone types (Table 3) was expected if seed mass is solely influenced by selection exerted by red squirrels: only serotinous cones experienced selection for small kernel size (Table 2). Finally, based on the lower survival rates of non‐serotinous than serotinous cones (medians: 0.667 and 0.886, respectively; Kruskal–Wallis test, *χ* ^2^ = 10.8, *p* = .001), the subpopulation having non‐serotinous cones should experience stronger selection than those having serotinous cones, but that was not the case (Table 2). The lower survival rate of non‐serotinous cones could have been an artifact of open cones being more susceptible than closed cones to being dislodged during winter and spring storms. However, this alternative is inconsistent with the absence of a reduction in cone persistence with increasing time after cones had opened (non‐serotinous cone survival did not differ between trees re‐photographed in October 2017 versus May 2018; mean ± *SE*: 0.46 ± 0.10 and 0.63 ± 0.04, respectively; *t* test, *t* = 1.46, *df* = 15.1, *p* = .16).

**TABLE 3:** Traits of cones (mean ± *SE*, untransformed) from trees having either serotinous or non‐serotinous cones

Trait	Serotinous cones	Non‐serotinous cones	*p*‐value
Scale width (mm)	4.65 ± 0.13	4.56 ± 0.15	.558
Cone length (mm)	34.99 ± 0.57	35.62 ± 0.58	.452
Cone width (mm)	22.69 ± 0.39	22.86 ± 0.37	.745
Cone width to length ratio	0.651 ± 0.009	0.644 ± 0.007	.581
Cone mass (g)	4.675 ± 0.154	4.857 ± 0.152	.428
Individual seed kernel mass (mg)	2.78 ± 0.10	2.87 ± 0.09	.440
Number of full seeds	15.0 ± 1.0	21.9 ± 1.0	<.001
Kernel mass to cone mass ratio	0.010 ± 0.001	0.015 ± 0.001	<.001
Sample size (number of trees)	62	67	

Note

*p*‐values based on *t* tests of ln‐transformed data comparing the two cone types.

## 4 DISCUSSION

Red squirrels tended to preferentially harvest cones with regard to the same traits for both cone types, but trees with serotinous cones tended to experience stronger selection than did trees with non‐serotinous cones. Traits for which trees with serotinous cones tended to experience stronger negative selection were the ratio of kernel mass to cone mass, individual kernel mass, and the number of full seeds per cone (Table 2; Figure 3). Consistent with evolution in response to these differences in the intensity of selection, serotinous cones had a lower ratio of kernel to cone mass (Table 3). In contrast, there was little difference in kernel mass between cone types even though selection for smaller seed size occurred only for seeds in serotinous cones (Table 2). Undoubtedly, kernel mass is also affected by selection at other times such as during and after germination (e.g., Salazar‐Tortosa, Castro, Saladin, Zimmermann, & Rubio de Casas, 2020; Tiscar Oliver & Lucas Borja, 2010), and such selection could account for the similarity of kernel size between the two cone types. The large difference in the number of seeds per cone between the two cone types (Table 3) is seemingly greater than expected by the relatively slight differences in their associated selection differentials (Table 2). However, selection strength is also influenced by the predictability of relative fitness from its relationship to the trait (Brodie & Brodie, 1999). A measure of predictability is the correlation coefficient of the relationship. By this measure, the relationship between relative fitness and seed number for trees with serotinous cones was more predictable than for those with non‐serotinous cones (*r *= −.456 and −.348, respectively).

Although higher levels of predation act to cause stronger selection (Benkman, 2013), we observed stronger selection on serotinous cones than on non‐serotinous cones despite lower overall predation on serotinous cones. More intense selection on serotinous than non‐serotinous cones likely occurs for two reasons. First, red squirrels have less time to assess and harvest non‐serotinous cones than serotinous cones, which could lead to less accurate assessment of cone value from trees with non‐serotinous cones. Red squirrels harvest and cache cones mostly after seeds mature and before cones begin to open (Smith, 1968, 1970), and presumably assess cone value by foraging on cones and perhaps by using visual and olfactory cues. Although the seeds of both cone types mature at the same time in late summer, non‐serotinous cones open several weeks later, whereas those of serotinous cones usually stay closed for a decade or more (Critchfield, 1980; Koch, 1996). Several weeks may be insufficient for red squirrels to assess the value of cones from many trees, whereas the much longer time period available to assess serotinous cones should allow red squirrels to more accurately assess cone differences among trees and harvest cones accordingly. Second, Smith (1970) showed that a consumer gains more in terms of a reduction in the proportion of the day spent foraging by being selective among less valuable food types than among similar differences in more valuable food types. Because non‐serotinous cones are on average more valuable than serotinous cones to red squirrels, it may be advantageous for squirrels to initially and less selectively harvest the more valuable cones (mostly non‐serotinous cones) during the several week window when non‐serotinous cones are mature and closed, and then be increasingly selective when harvesting the remaining closed and less valuable serotinous cones.

In contrast to Talluto and Benkman (2014), we found that red squirrel predation was higher on non‐serotinous cones than on serotinous cones. We note that Talluto and Benkman (2014) measured predation by red squirrels during both the first and subsequent years for serotinous cones, but even over the first year, which is what we measured, they found predation on serotinous cones was higher. Intriguingly, Talluto and Benkman (unpublished data) found that the ratio of kernel to cone mass for serotinous cones averaged 0.0125 (±0.0005, *n* = 178 trees) in Yellowstone National Park, which is larger than what we found for serotinous cones (0.0099; Welch's ANOVA, *F*_1,120.9_ = 7.65, *p* = .007). This difference in apparent seed defense is consistent with the hypothesis that stronger selection for seed defenses in serotinous cones could lead to, as we have apparently found in our study area, elevated defenses (lower ratio of kernel to cone mass) that act to reduce predation on serotinous cones and thus relax selection against serotiny by red squirrels.

Even though we favor the hypothesis that higher levels of defense in serotinous cones at our study site accounts for the lower levels of predation on serotinous cones in our study than in Talluto and Benkman’s (2014) study, differences in the proportion of trees that have serotinous cones could provide an alternative explanation. For example, as the proportion of trees in a territory that have non‐serotinous cones increases, red squirrels could become saturated in their ability to harvest a high proportion of the less defended (more valuable) non‐serotinous cones during the several weeks before they open. Conversely, a high proportion of trees with serotinous cones could lead to relatively higher predation on non‐serotinous cones. If the proportion of trees with serotinous cones was higher in our study than in that of Talluto and Benkman (2014), then this might account for the higher relative predation rate on non‐serotinous cones that we found. We are unable to compare the levels of serotiny between the two studies. However, we can examine whether non‐serotinous cone survival decreased with an increase in the proportion of trees with serotinous cones on a squirrel territory. In contrast to this expectation, non‐serotinous cone survival was not related to the territory‐level frequency of serotiny (*r* = 0.165, *p* = .183; the proportion of trees that were serotinous on the 14 territories ranged between 0.05 and 0.75, with an overall mean of 0.32). In fact, non‐serotinous cone survival tended to increase as the frequency of trees with serotinous cones increased, the opposite predicted if variation in their relative abundances contributed to the differences between the two studies.

In conclusion, our results show the potential for the interaction between selection from both fire and seed predation by red squirrels, and the evolutionary outcome of this interaction, to be altered by the evolution of seed defenses in response to selection by red squirrels. Increasing fire frequency and a reduction in the occurrence of red squirrels favor an increase in the frequency of serotiny (Benkman & Siepielski, 2004; Schoennagel et al., 2003; Talluto & Benkman, 2014). However, selection against serotiny by red squirrels is dependent on the extent to which serotinous cones are disproportionately harvested (Talluto & Benkman, 2014). If serotinous cones have elevated defenses, this might deter red squirrels and reduce predation rates, relaxing selection against serotiny. This may have occurred in our study population, potentially allowing higher levels of serotiny to evolve with tremendous community and ecosystem consequences (Turner et al., 2003, 2016). However, it is unknown why defenses might be more elevated in the subpopulation of serotinous individuals in our study area than where Talluto and Benkman (2014) conducted their study. Finally, our results indicate that it would be worthwhile to examine the mechanism and extent to which traits determining thresholds for cone opening (serotiny) are linked to those related to deterring the harvest of cones by red squirrels.

## CONFLICT OF INTEREST

None declared.

## AUTHOR CONTRIBUTIONS

**Anna L. Parker:** Conceptualization (supporting); data curation (lead); formal analysis (lead); investigation (lead); methodology (supporting); resources (equal); writing–original draft (equal); writing‐review and editing (equal). **Craig Benkman:** Conceptualization (lead); formal analysis (equal); funding acquisition (lead); investigation (supporting); methodology (equal); supervision (equal); writing–original draft (equal); writing‐review and editing (equal).

## Data Availability

All the data used in the analyses are available from the Dryad Digital Repository (https://doi.org/10.5061/dryad.hdr7sqvf7).
